# Defect Detection of Composite Material Terahertz Image Based on Faster Region-Convolutional Neural Networks

**DOI:** 10.3390/ma16010317

**Published:** 2022-12-29

**Authors:** Xiuwei Yang, Pingan Liu, Shujie Wang, Biyuan Wu, Kaihua Zhang, Bing Yang, Xiaohu Wu

**Affiliations:** 1 Institute of Automation, Qilu University of Technology (Shandong Academy of Sciences), Jinan 250014, China; 2 Key Laboratory of Microwave Remote Sensing, National Space Science Center, Chinese Academy of Sciences, Beijing 100190, China; 3 University of Chinese Academy of Sciences, Beijing 100049, China; 4 Qingdao Quenda Terahertz Technology Co., Ltd., Qingdao 266100, China; 5 Basic Research Center, School of Power and Energy, Northwestern Polytechnical University, Xi’an 710072, China; 6 Shandong Institute of Advanced Technology, Jinan 250100, China; 7 Henan Key Laboratory of Infrared Materials & Spectrum Measures and Applications, School of Physics, Henan Normal University, Xinxiang 453007, China; 8 Centre for Advanced Laser Manufacturing (CALM), School of Mechanical Engineering, Shandong University of Technology, Zibo 255000, China

**Keywords:** defect detection, composite material, terahertz image, faster R-CNN

## Abstract

Terahertz (THz) nondestructive testing (NDT) technology has been increasingly applied to the internal defect detection of composite materials. However, the THz image is affected by background noise and power limitation, leading to poor THz image quality. The recognition rate based on traditional machine vision algorithms is not high. The above methods are usually unable to determine surface defects in a timely and accurate manner. In this paper, we propose a method to detect the internal defects of composite materials by using terahertz images based on a faster region-convolutional neural networks (faster R-CNNs) algorithm. Terahertz images showing internal defects in composite materials are first acquired by a terahertz time-domain spectroscopy system. Then the terahertz images are filtered, the blurred images are removed, and the remaining images are enhanced with data and annotated with image defects to create a dataset consistent with the internal defects of the material. On the basis of the above work, an improved faster R-CNN algorithm is proposed in this paper. The network can detect various defects in THz images by changing the backbone network, optimising the training parameters, and improving the prior box algorithm to improve the detection accuracy and efficiency of the network. By taking the commonly used composite sandwich structure as a representative, a sample with typical defects is designed, and the image data are obtained through the test. Comparing the proposed method with other existing network methods, the former proves to have the advantages of a short training time and high detection accuracy. The results show that the mean average precision (mAP) without data enhancement reached 95.50%, and the mAP with data enhancement reached 98.35% and exceeded the error rate of human eye detection (5%). Compared with the original faster R-CNN algorithm of 84.39% and 85.12%, the improvement is 11.11% and 10.23%, respectively, which demonstrates superb feature extraction capability and reduces the occurrence of network errors and omissions.

## 1. Introduction

Composite structures are widely used in aerospace, automotive, shipbuilding, and other fields thanks to their advantages, such as low specific gravity and good fatigue resistance. Therefore, composite materials play a very important role in the development of modern science and technology. However, in the process of composite material preparation, defects such as delamination, debonding, voids, and inclusions will inevitably occur because of the process, environment, and other factors. It is crucial to detect these defects to avoid certain hazards. The currently available general inspection means are ultrasonic inspection [1], infrared nondestructive testing [2], X-ray, thermal wave detection [3], etc. Nevertheless, these detection methods cannot correctly and clearly detect defects in composite materials.

Terahertz time-domain spectroscopy imaging, as a noncontact, penetrating nondestructive testing (NDT) technique, can characterise nonmetallic materials with multilayer structures [4,5,6]. Compared with the ultrasonic testing technology, the terahertz NDT technology does not need to use a coupling agent in the testing process, which reduces the pollution on the surface of the tested object. Compared with X-ray, THz wave has less energy and no ionisation damage to the human body and the detected object, so it offers more safety. Compared with NIR technology, THz technology has a strong anti-interference ability, and THz waves can penetrate nonpolar and nonmetallic materials such as clothing and paper boxes [7]. THz wave is transient in the time domain, which can extract the depth and thickness data of materials, and broadband in the frequency domain, which can reflect the difference of substances in the spectrum and be used to identify substances. Therefore, THz imaging technology for the defect detection of substances has great application potential.

In 2015, Guo et al. [7] used a THz-TDS system and a return oscillator continuous terahertz wave system to detect delamination, intercalated metal, and thermal damage defects in glass fibre composites. In 2019, Zhang et al. [8] performed defect detection on composite materials using terahertz reflectance laminar imaging and compared it with the B-scan imaging method. Yet the diffraction effect of terahertz waves can lead to the blurring of image edges, and the interference effect can also lead to the presence of significant bright and dark streaks in the image. Terahertz images can also be affected by factors such as the environment, which can lead to suboptimal image quality [9]. It comes with great subjective interference on the manual recognition of defects in terahertz images, and there will be a misjudgement.

Defect detection methods based on deep learning are classified into single-stage and two-stage. The main single-stage networks are SSD [10], YOLO [11,12,13,14,15] series networks, and so on. The main two-stage networks are R-CNN [16], fast R-CNN [17], faster R-CNN [18], mask R-CNN [19], and so on. In 2019, Hou et al. [20] proposed an image-detection algorithm based on an improved faster R-CNN, which obtained a high accuracy and detection rate. In 2022, Xue et al. proposed the mask-CGANs model and used the RetinaNet detection network [21] to build a conditional generative adversarial network used for the accurate segmentation of terahertz images [22].

In this paper, a glass fibre composite sample with three kinds of embedded defects is designed, and the terahertz image of the sample is obtained by scanning imaging with the terahertz time-domain spectral system. Then, an improved faster region-convolutional neural network (faster R-CNN) is proposed to realise the intelligent recognition of THz images. By comparing the detection results with those of two existing detection networks, the accuracy is improved by 11.11% and 10.23%, respectively, which proves that the proposed method can effectively reduce missed detection. THz-NDT technology is developing in a real-time and intelligent direction, so it is of great significance to explore new intelligent detection methods to improve detection efficiency.

## 2. Principles and Methods

### 2.1. Faster R-CNN

Faster R-CNN is a two-stage network model first proposed by Ross B. Girshick in 2016. The first stage obtains the candidate frames by feature extraction using the backbone network, and the second stage classifies the contents of the candidate frames. The commonly used backbone networks are VGG [23], ResNet [24], Xception [25], etc., and the selection of one has a crucial impact on detection accuracy. The algorithm has the characteristics of fast operation speed and high detection accuracy, so it is widely used to detect targets in images and more. In this section, the overall architecture of faster R-CNN is illustrated and introduced using ResNet as the backbone extraction network.

**Figure 1:**
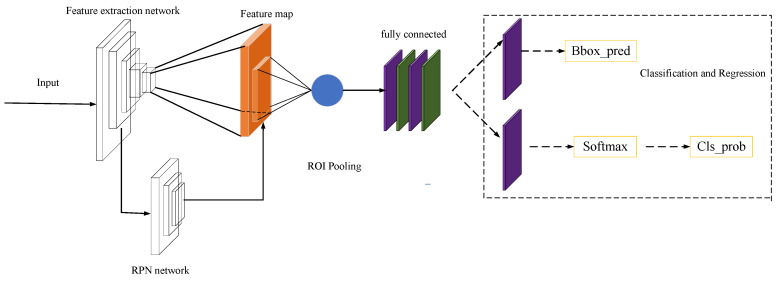
Faster R-CNN network framework diagram.

As shown in Figure 1, the faster R-CNN network can be divided into four parts:

(1) Feature extraction network. The feature extraction of the input image mainly uses convolutional neural network to obtain the feature map of the image.
(2) Candidate region generation network RPN (region proposal network). It is used to generate candidate regions where detection targets may exist. A more accurate detected region is obtained by classifying and regressing the predefined anchor frames on the feature map obtained in the previous step. RPN can improve the efficiency of candidate region selection and greatly reduce network time consumption.
(3) ROI (region of interest) pooling. On the one hand, the corresponding feature vectors are extracted for the candidate regions. On the other hand, the feature maps corresponding to the candidate regions are adjusted to a fixed size to facilitate subsequent accurate classification.
(4) Classification and regression. Softmax is used to classify the feature vectors to determine the categories. Then the exact position is selected for the detection box by using bbox_pred.

The Softmax function is shown below.

(1) $$y_{k}=\frac{e^{a_{k}}}{\sum_{i=1}^{n}e^{a_{i}}}$$

where a represents an array, $a_{k}$ represents the *k*’th element in the array *a*, and $y_{k}$ represents the Softmax value of the *k*’th element.

### 2.2. The Improved Faster R-CNN

The improved faster R-CNN network consists mainly of three parts: an improved backbone network, resetting the anchor frame of the dataset, and the Bayesian optimisation of hyperparameters.

(1) Backbone network improvement

**Figure 2:**
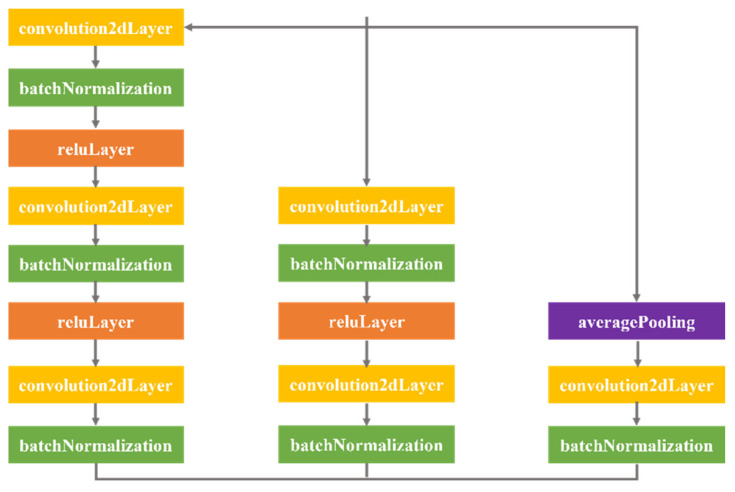
Improved residuals module.

The original faster R-CNN algorithm uses VGG16 in the backbone network feature extraction module to complete the image feature extraction [26]. To extract more comprehensive features of the composite sandwich structure, the Resnet50 network, which can extract image detail information, is selected for the feature extraction of the composite sandwich structure in this paper. In addition, in the detection of defects in composite sandwich structures, there are currently two problems: defects occupy a small area of the whole image, and a small percentage of information is obtained. To avoid the redundancy of useless information, this paper adds another layer of 2 × 2 average pooling (avg-pool) to integrate spatial information before downsampling the convolution kernel of the residual module, which can prevent overfitting in this layer. In addition, to enhance the perceptive field of the network with respect to the input features and to more thoroughly extract the low-level detail information and high-level semantic information of the input image, a layer of modules containing convolution, batch normalisation, and linear correction is built into the residual network. Compared with the faster R-CNN network model, the improved residual structure of the algorithm extracts more information from the input feature layer and makes the network more expressive. The improved residuals module is shown in Figure 2.

(2) Anchor boxes for resetting datasets

① Anchor box

Anchor box estimation is a classic concept in target detection, providing faster detection and solving multiscale target problems. To avoid the problem of an imbalance between positive and negative samples due to the use of generic anchor frames in the dataset of this paper, this paper proposes an improved Kmeans algorithm for estimating anchor frames and reclustering the dataset to obtain a priori frames suitable for detecting the dataset of this paper.

② Improved Kmeans algorithm

**Figure 3:**
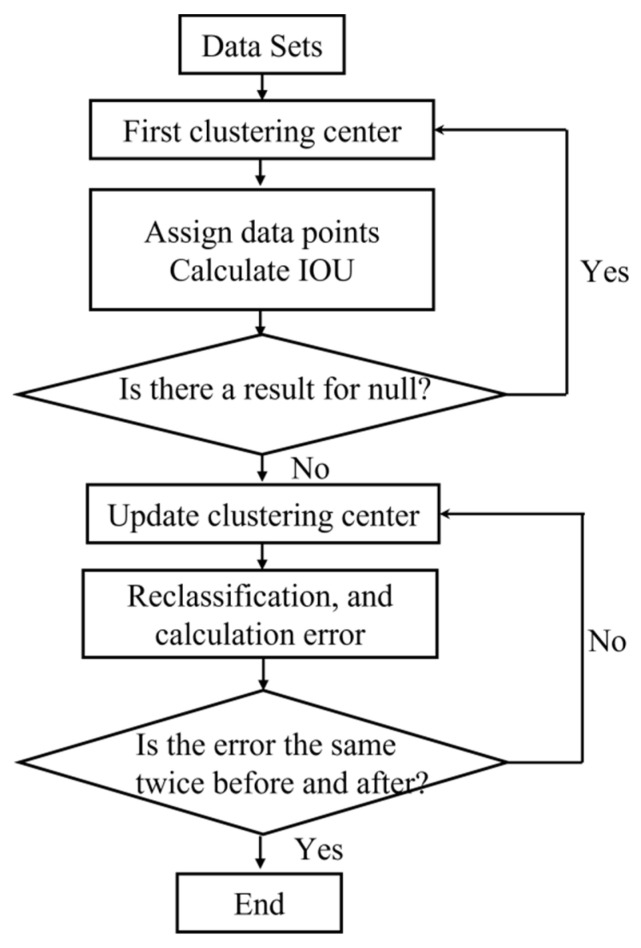
Flowchart of improved Kmeans clustering algorithm.

The detection targets in this paper take the form of circles, triangles, and quadrilaterals. Most of the a priori boxes generated by clustering of the dataset are of similar size and similar aspect ratio. To make the improved faster R-CNN algorithm quickly and accurately predict the location of the target more, an improved Kmeans process is used for selecting the nine cluster-class centres one by one, as shown in Figure 3.

(3) Bayesian optimisation network training hyperparameter

This hyperparameter is for the convolutional neural network in the network training process for the problem of inefficiency and poor accuracy. This paper uses Bayesian algorithms to optimise the training hyperparameters of the network. Bayesian optimisation is an algorithm that uses Bayes to search for the optimal value of an objective function, where the probabilistic agent model and the collection function are the core parts of the Bayesian optimisation algorithm. Currently, the commonly used probabilistic agent model is a Gaussian process, and the collection function consists of the posterior probabilities of the objective function. To minimise the total loss *r*, the Bayesian optimisation algorithm selects the evaluation point *x_i_* by using a collection function. The process can be expressed as follows:

$$x_{i+1}=\underset{x\in X}{\max}\lambda (x,D_{1:i})$$

(2) $$r_{i}=\left|y^{*}-y_{i}\right|$$

where *x* is the decision space, $\lambda (x,D_{1:i})$ is the collection functions, and y* is the optimal solution.

The Bayesian optimisation algorithm is implemented by the following steps:

① Determine the maximum number of iterations N.
② Use the collection function to obtain the evaluation point *x_i_*.
③ Evaluate the objective function value *y_i_* by using the evaluation point *x_i_*.
④ Update the probabilistic proxy model after integrating data *Dt*.
⑤ Return to step ② and continue iterating if the current number of iterations n is the maximum number of iterations N; otherwise, output *x_i_*.

### 2.3. Evaluation Indicators

The overall mean average precision (mAP) is used as an evaluation index for detection accuracy according to detection accuracy and speed requirements.

Overall average accuracy

(1) Recall and precision

Recall is the proportion of all positive samples in the test set that are correctly identified as positive. Precision is the percentage of positive samples that are identified in the number of images identified as positive samples. The expressions for recall and precision are as follows:

(3) $$\left\{ \begin{array}{l} \mathrm{Recall}=\frac{\mathrm{TP}}{\mathrm{TP}+\mathrm{FN}}\\ \mathrm{Precision}=\frac{\mathrm{TP}}{\mathrm{TP}+\mathrm{FP}} \end{array}\right.$$

where TP represents the number of positive samples correctly identified as positive, FN is the number of positive samples incorrectly identified as negative, and FP is the number of negative samples incorrectly identified as positive.

(2) Average precision (AP)

AP is an important indicator to evaluate the accuracy of a target detection model for a class and can be reflected by the PR curve. The PR curve combines the detector’s ability to perform correct classification (precision) with the ability to find all relevant objects (recall). When the area of the PR curve is larger, the accuracy of the model’s localisation and recognition is higher. AP can be expressed as follows:

(4) $$AP=\int_{0}^{1}p(r)dr$$

(3) mAP

In the case of composite sandwich structure inspection targets, mAP is the mean value of the average accuracy of the category target, which is expressed as follows:

(5) $$\left|\mathrm{mAP}=\frac{\sum_{k=1}^{n}{\mathrm{AP}}_{k}}{n}\right|$$

where *n* is the number of target category and k is a specific category.

## 3. Experiments and Equipment

### 3.1. Preparation of Samples

**Figure 4:**
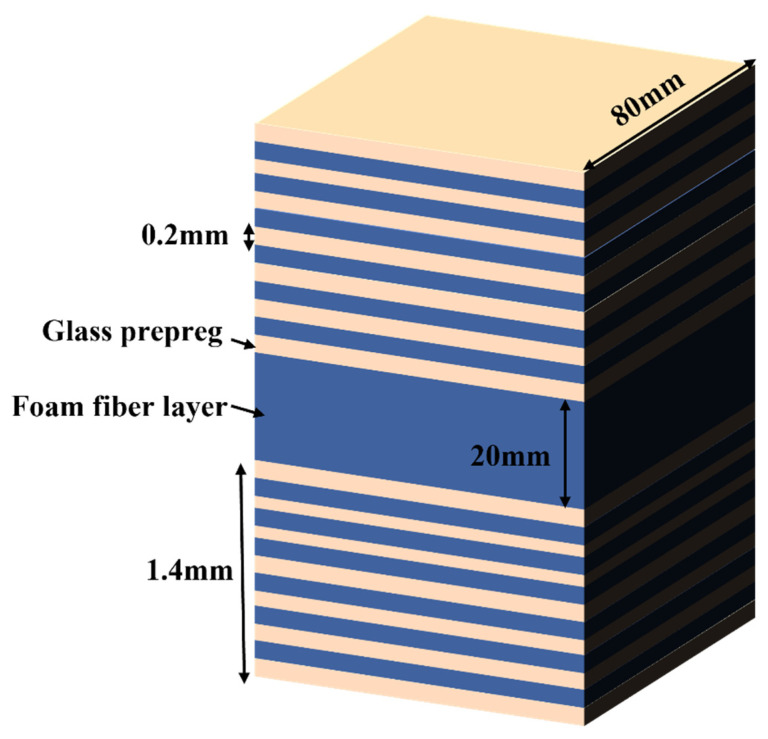
Design of the sandwich structure.

A composite multilayer structure for aircraft radomes is selected for this work. The structure consists of upper and lower skins, adhesives, and intermediate core materials. The skin is made of glass fibre material with good wave-transparent performance, the thickness is about 1.4 mm (7 layers), and the laying angle is (0°/90°/45°/−45°). The intermediate core material is the most commonly used PMI foam in aerospace structures. FOAM, an MH-type PMI foam, is selected as the core material. The design diagram is shown in Figure 4.

### 3.2. Artificial Defect Preset

The main defects of the composite sandwich structure are delamination, debonding, and cavity, which are distributed at different depths of the sandwich structure. The defect shapes and sizes were laid out to verify the detection ability of the proposed improved faster R-CNN network for defects. In addition, to verify the ability of the neural network to detect the defect depth, the location of the defect depth is laid out. The defect types contain the follows:

(1) Delamination defects. In the process of glass fibre material prefabrication, the delamination defect is represented by adding polytetrafluoroethylene (PTFE) flakes in between the middle of the third and fourth prepreg layers. Because the refractive index of PTFE is close to that of air, it can replace the delamination effect with the thickness of 0.2 mm.
(2) Debonding defects. When the glass fibre material is glued to the foam, a PTFE sheet is placed. It can replace the state without gluing, and the thickness of a PTFE sheet is 0.2 mm.
(3) Hollow defects. This involves the setting of cavities of different sizes, shapes, and depths on the surface of the foam.

**Figure 5:**
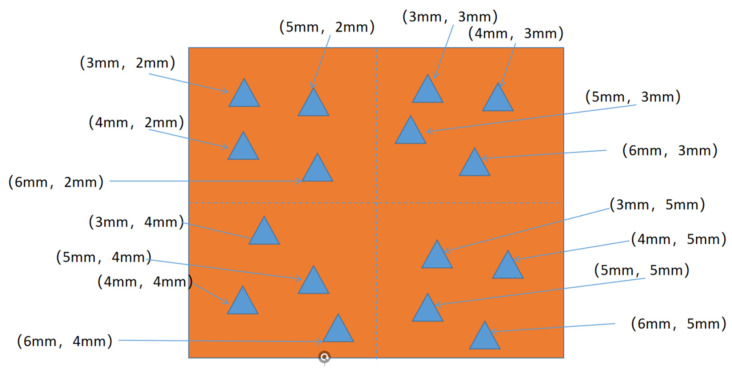
Top view of the defective sample.

A large number of sample data are required for network training, so samples of datasets for network training and validation were first prepared. Samples A1 to A8 were used to improve the training and validation of the faster R-CNN network. Three types of defects were randomly preset at different depth positions within these samples. The defect shapes were triangle, circle, heterotropic, and other shapes. Figure 5 shows the design drawing of one of the training set samples.

**Figure 6:**
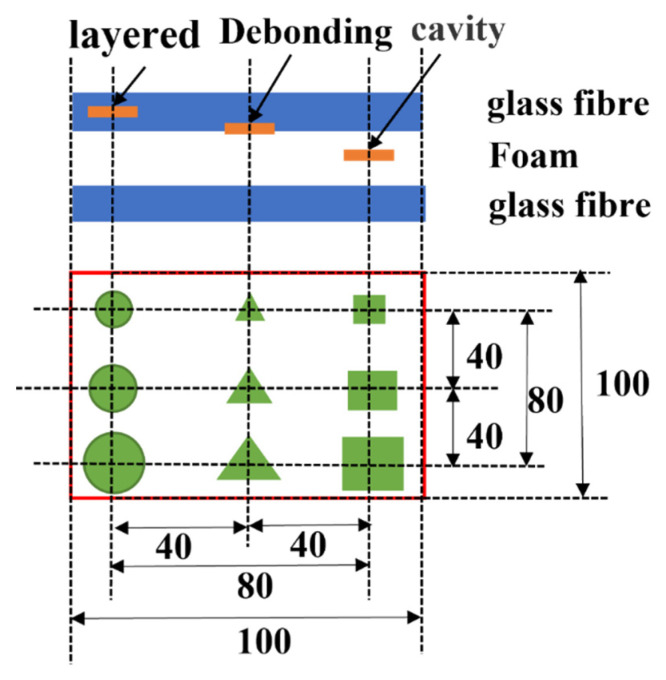
Three types of defect distributions.

In the sample for testing the network, layered defects used thin circular sheets of 4 mm, 3 mm, and 2 mm diameter PTFEs with a thickness of 0.2 mm. The debonding defect is placed in the equilateral triangular sheet of PTFE, which is also a substitute for the nonadhesive state. The PTFE sheets are 4 mm, 3 mm, and 2 mm in side length and 0.2 mm in thickness. For void defects, square holes with the same depth and different side lengths were punched in the foam, with side lengths of 4 mm, 3 mm, and 2 mm and a depth of 2 mm. A sample design diagram with three defect types is shown in Figure 6.

**Figure 7:**
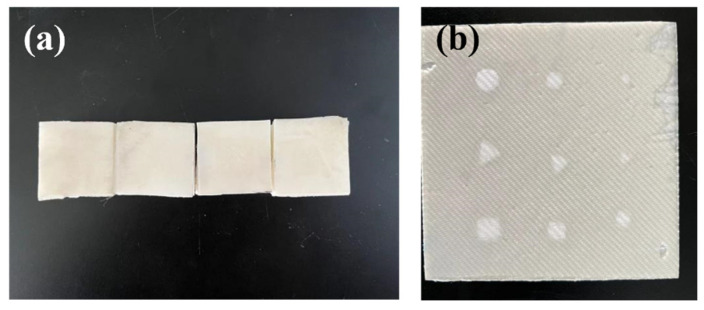
Physical picture of the sample: (**a**) part of the training set samples and (**b**) a sample of the test network.

**Figure 8:**
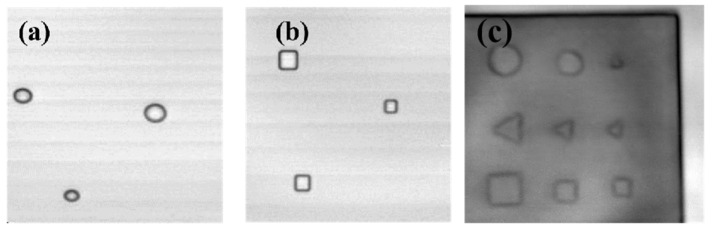
Defect types. (**a**) Terahertz image of sample A1 (**b**) Terahertz image of sample A2 (**c**) Terahertz image of test set sample.

The preparation process is to first carry out the lay-up of prepreg to prepare for glass fibre composites. Then the glass fibre is glued to the foam using an adhesive. Compaction is carried out by vacuum evacuation. The preparation is carried out in accordance with Q/MLPS1 “Hot Pressed Can Process for Fiber Reinforced Composites”. In order to prevent the collapse of the void defect during the vacuum-pressing process, the vacuum pressure is reduced to half of the normal value. Finally, high-temperature curing and cooling demoulding are performed. The actual processed samples are shown in Figure 7. Figure 7a is the sample of the training set, Figure 8b is a part of the validation set, and Figure 7b is the sample physical diagram of the test network. The image obtained using THz-TDS scanning imaging is shown in Figure 8.

### 3.3. THz-TDS Experimental System

**Figure 9:**
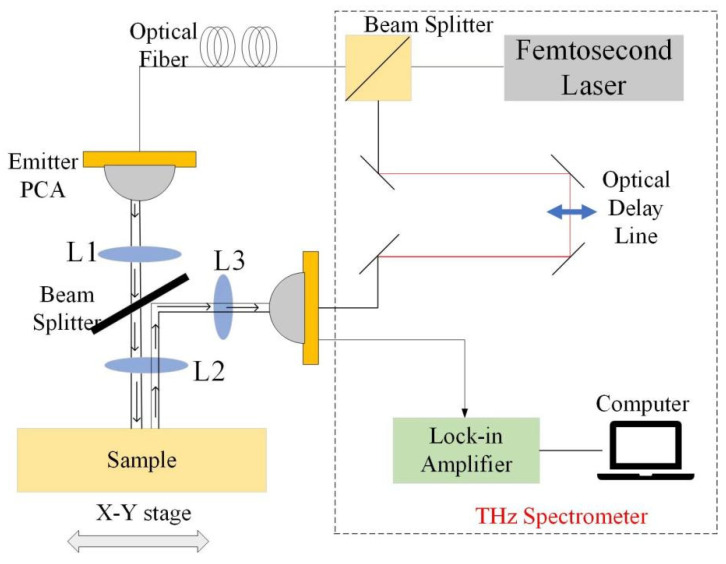
Schematic diagram of the THz-TDS system.

The reflection mode of the terahertz time-domain spectroscopy system selected in this paper is shown in Figure 9. The experimental setup includes an ultrafast femtosecond laser, an optical delay line, a transmitter and a receiver, photoconductive antennas (PCAs), lock-in amplifiers, and a computer for controlling the device and processing the signals. A 2 mm thick silicon wafer in the experimental setup acts as a beam splitter to guide the reflected terahertz beam from the sample to the receiver. The emitted terahertz pulses are collimated and focused by a TPX lens to reach the surface of the sample to be measured. After the photoelectric effect has been generated, it is collimated and focused by a symmetrical TPX lens to the fibre-coupled terahertz receiver. It is then fed to the fibre femtosecond laser via a fibre delay line system. At the detection end, the THz pulse is obtained by electro-optical sampling. Then the THz signal is obtained by equivalent sampling, which in turn collects the signal through the acquisition system and processes and displays the signal through the upper computer system. The system samples time signals with a step size of 0.01 ps, a time-domain scan length of 120 ps, a pulse width of 1ps, and a modifiable scan speed of up to 60 Hz. To avoid the influence of temperature and humidity on the system performance, the relative humidity of the test environment was maintained at 25%, and the temperature was maintained at 25 °C.

### 3.4. Data Acquisition and Preprocessing

In this paper, the reflection mode of the terahertz time-domain spectral system (mentioned above) and the two-dimensional scanning platform were used to scan and image the samples. Through experimental comparison, the scanning step of the scanning platform was finally set as 0.5 mm. To increase the number of training datasets and improve the robustness of network training, time-domain maximum imaging and specific frequency amplitude imaging with obvious difference characteristics were selected to ensure that every defect in the image can be presented. The collected and selected datasets were preprocessed to train the improved faster R-CNN network in this work. The initial dataset of 300 images was first processed one by one using the superresolution reconstruction method to make the image signal–to-noise ratio higher. To increase the number of datasets, each image was cropped into four copies. Then the cropped images were subjected to random rotation, contrast adjustment, brightness adjustment, and other operations for data enhancement. The images with poor results were selected again, and finally 1300 images of the dataset were obtained, including 1000 images of the training set and 300 images of the test set. Finally, the defects in the training set images were manually annotated using the image labelling function in the MATLAB software. The annotated data were imported into the workspace in “.groundTruth” format. Then they were stored in the folder in “.mat” format.

The parameters used were set in the training process. The initial learning rate was set to 0.01, the BatchSize was set to 4, and the maximum number of rounds maxEpochs was set to 1000. The learning rate adjustment strategy decreased as the Epoch increased, using a linear increase to change the learning rate.

## 4. Results and Discussions

### Result of Resetting Dataset Anchor Box

The algorithm in this paper uses the intersection ratio of the cluster-class centre prior frame to the area of other prior frames as the metric distance to calculate the intersection over union (IoU).

Usually, the IoU of the coincidence of the predicted frame and the actual annotation frame is greater than 0.5, and the target can be considered to be successfully detected. The IoU is used to characterise whether the anchor frame parameters are optimal. The training dataset is reclustered to generate nine new sets of anchor frame parameters: (22,26), (3,12), (22,16), (27,28), (9,16), (25,21), (19,20), (16,15), and (15,27). The average IoU obtained for the training data is 96.34%

The hyperparameters, such as learning rate, were optimised using Bayesian optimisation; the maximum number of iterative rounds was set to 100; and the acquisition function was expectation boosting. The decision space is shown in Table 1.

After the Bayesian optimisation of the hyperparameters, it is found that the learning rate is 0.001679, the momentum parameter is 0.8157, and the L2 regularisation is 2.368 × 10^−5^. The SGDM optimisation algorithm is experimentally analysed to outperform both RMSProp and Adam optimisers in terms of training speed and prediction accuracy. This is because the SGDM optimisation algorithm introduces a momentum factor to update the model parameters while selecting a portion of the sample data for training each time.

Improved faster R-CNN network performance analysis

**Figure 10:**
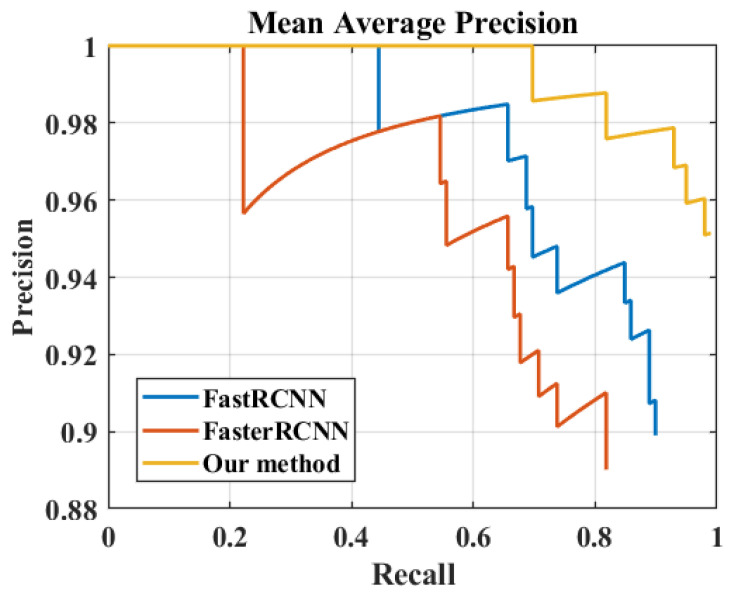
P-R curves of the three networks.

To test the superiority of the improved faster R-CNN against defects, the results of the faster R-CNN and those of the improved faster R-CNN are compared for the same parameter settings. The ResNet50 network is the backbone of all three of these networks. As shown in Figure 10, the P-R curves of the three networks are plotted. The horizontal axis is the recall rate, and the vertical axis is the precision. The average precision value can be calculated from the area enclosed by the curve. It can be seen that the improved network has a larger area and therefore a higher average precision value. The average accuracies obtained from the calculations are compared in Table 2. The average accuracy of the fast R-CNN without data enhancement preprocessing is 76.52%, and the average accuracy of the original faster R-CNN is 84.39%. The average accuracy of the improved faster R-CNN in this study is 95.50%, which is 18.98% and 11.11% better than the other two networks, respectively. After data enhancement preprocessing, the average accuracy of the fast R-CNN is 79.04%. The average accuracy of the original faster R-CNN is 88.12%. The improved faster R-CNN in this study has an average accuracy of 98.35%, which is 19.31% and 10.23% better than the other two networks, respectively. One can see that the detection accuracy of the faster R-CNN network improved by this study is higher than other networks, both without data augmentation and after data augmentation.

**Figure 11:**
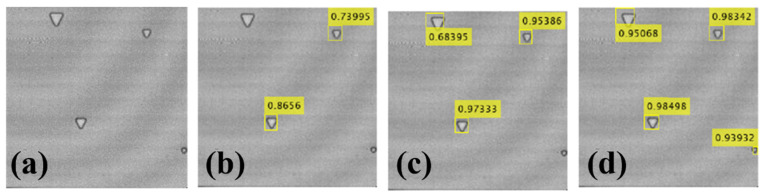
Comparison of defect detection performance of the validation set: (**a**) original, (**b**) fast R-CNN, (**c**) faster R-CNN, and (**d**) improved faster R-CNN.

To further analyse the detection ability of the improved faster R-CNN network, images in the validation set and in the test set are selected, respectively. The validation set contains a defect triangle with a minimum side length of 2mm, and the accuracy of the three networks is compared. Figure 11a shows the imaging results of a test set image obtained by using time-domain maximum imaging; that is, the maximum value of the time-domain signal species of each scanning point is selected as the pixel value of the image. Figure 11b–d respectively show the results of defect detection using different methods, where the location and accuracy of the detected defects are marked. In terms of the number of detections, it can be seen from Figure 11b,c that the fast R-CNN method misses two defects, while the faster R-CNN misses one defect with the smallest detection rate. The proposed network structure can detect all four defects in the image. In terms of detection accuracy, for each defect, the detection accuracy of the proposed network structure is higher than that of the other two networks.

**Figure 12:**
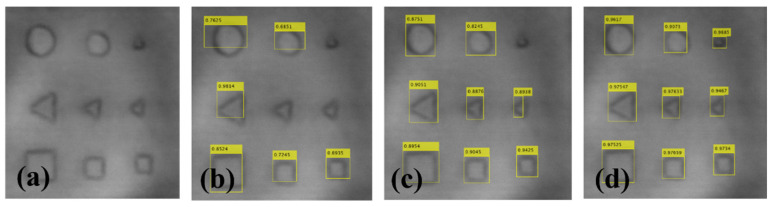
Comparison of test image defect detection performance: (**a**) original, (**b**) fast R-CNN, (**c**) faster R-CNN, and (**d**) improved faster R-CNN.

Figure 12a shows the imaging results of the validation set samples obtained by using time-domain maximum imaging; that is, the maximum value of time-domain signal species of each scanning point is selected as the pixel value of the image. The defect edges in the image are blurred. Figure 12b–d respectively show the defect recognition using three methods for the image. In the fast R-CNN, the detection of defects is easy to miss with small size and fuzzy edges, and the detection rate of the detected defects is also low. The faster R-CNN can detect most defects, except for the smallest and most ambiguous defects, whereas the improved faster R-CNN proposed in this paper can detect all defects, and its detection accuracy is the highest.

One can see that fast R-CNN misses two defect targets. The faster R-CNN misses the smallest defect target. The improved faster R-CNN not only detects all the defects but also has the highest accuracy for each defect. The fast R-CNN and faster R-CNN methods are prone to missing the detection of small defects and blurred defects. The improved faster R-CNN detected all the defects, which fully demonstrated the detection capability of the improved faster R-CNN for various defects.

## 5. Conclusions

In summary, we proposed a defect detection method based on the faster R-CNN algorithm for defects in the composite sample parts. Defect detection experiments were performed on the samples by building a terahertz time-domain spectroscopy system. The target detection experimental dataset was obtained by preprocessing, such as data screening, data enhancement, and defect labelling. The anchor box was optimised by the Kmeans clustering algorithm to improve detection accuracy and detection speed by a small margin. The feature fusion of feature maps in the path aggregation network was adjusted to make full use of the shallow details. The target feature detection layer was built on the new three scales. The improved faster R-CNN algorithm trains samples with 100 epochs. After several trials of training the network, an accuracy of 98% and a recall of 92.02% were obtained from the test set. It resulted in a 10.23% improvement over the original faster R-CNN algorithm and an 8.51% reduction in the leakage detection rate. The method proposed in this paper featured a large improvement in detecting small targets and eliminated almost all missed and wrong detections, implying that the model had better robustness. The detection speed was increased while the detection accuracy was guaranteed, and the model size was reduced by light-weighting the network to be used in embedded systems for industrial inspection.

## Figures and Tables

**Table 1:** Hyperparameters and their ranges of improved ResNet 50.

Hyperparameters	Minimum Value	Maximum Value
Initial Learn Rate	1 × 10^−4^	1
Momentum	0.8	0.99
L2 Regularisation	1 × 10^−5^	1 × 10^−2^

**Table 2:** Performance comparison of different networks.

Models	Backbone	Average Accuracy Value/%	
		Unused Data Enhancement	Data Enhancement
Fast R-CNN	ResNet50	76.52	79.04
Faster R-CNN	ResNet50	84.39	88.12
Improved Faster R-CNN	ResNet50	95.50	98.35

## Data Availability

Not applicable.
